# Chitosan in Sparkling Wines Produced by the Traditional Method: Influence of Its Presence during the Secondary Fermentation

**DOI:** 10.3390/foods9091174

**Published:** 2020-08-25

**Authors:** Antonio Castro Marín, Claudio Riponi, Fabio Chinnici

**Affiliations:** Department of Agricultural and Food Sciences, University of Bologna, Viale Fanin, 40, 40127 Bologna (BO), Italy; antonio.castromarin2@unibo.it (A.C.M.); claudio.riponi@unibo.it (C.R.)

**Keywords:** chitosan, sparkling wine, foamability, sensory

## Abstract

Chitosan is a polysaccharide admitted in winemaking as clarifying, antimicrobial and chelating agent. In addition, evidence about its antioxidant and radical scavenging activities have been recently reported in wine conditions. As an insoluble adjuvant, chitosan efficacy also depends on the duration of its contact with the matrix. In the case of sparkling wines obtained following the traditional method, for instance, the addition of chitosan before the secondary fermentation would permit a prolonged contact of the polymer with wine and yeast lees. However, information on the effects of this practice on final products is totally unknown. In this work, the addition of chitosan during the secondary fermentation of a traditional sparkling wine production method has been investigated for its effects on both the physicochemical and sensory characteristics of the resulting wine. After 12 months of “sur lie” maturation, chitosan was found to increase the protein and amino acid content of wines up to about 50% and 9%, respectively, with limited change of phenolics and organic acids. Volatile compounds, particularly esters, were increased as well, which was reflected by higher values for fruity character and aroma intensity after sensory tests. Foaming features, evaluated by sensory and physical measurements, were also positively affected.

## 1. Introduction

The traditional method for sparkling wines production is based on two consecutive alcoholic fermentations (AF). In a first step, the base wine is obtained by conventional white winemaking procedures. Next, selected yeasts and sugars (liqueur de tirage) are added to promote a second AF, carried out in sealed bottles, that results in the further formation of ethanol, dissolved CO_2_ and volatile compounds, as main products [1,2]. Once secondary fermentation ends, bottled wines are subjected to a prolonged period in contact with dead yeast lees [3] during which autolysis of yeasts cells occurs. This leads to the release of various intracellular components such as nitrogen compounds, polysaccharides and some volatiles like terpenic and higher alcohols that impact the organoleptic properties of sparkling wines [2,4]. Secondary fermentation also affects the foaming properties of final product since peptides, amino acids and polysaccharides released during autolysis may have a positive effect on height and persistence of the foam itself, further contributing to the overall perceived wine quality [5].

Chitosan is the deacetylated derivative of chitin, the second most abundant biopolymer in the earth, extracted from shellfish, insects and fungus. Its structure, mainly constituted of glucosamine and *N*-acetylglucosamine units, confers it a great versatility with respect to several applications in food industries and interesting features including metal chelation, film forming properties or antimicrobial capacity [6,7,8,9,10,11]. Since its authorization in winemaking for metal and protein removal (maximum dose level 1 g/L) and microbial stabilization (maximum dose level 0.1 g/L) [12], the use of chitosan has aroused great interest in oenology. Colangelo and coworkers [13], for instance, reported a significant improvement of wines stability to heat test performed after fining treatments with chitosan. Other researches demonstrated that the presence of chitosan during fermentation can enhance the production of some volatile esters such as isoamyl acetate and phenethyl acetate together with medium-chain fatty acids and respective ethyl esters [14].

Chitosan can also act as an antioxidant in wine by means of various mechanisms, such as direct hydroxyl radical scavenging, prevention of the formation of 1-hydroxyethyl radical and metal chelation [15,16].

In principle, the traditional method for sparkling wine production could favour the action of chitosan as it permits both the presence of the polysaccharide during alcoholic fermentation and a prolonged permanence in the medium followed by a complete removal, as insoluble matter, during the degorgement step. However, information on the effects of the addition of chitosan during the prise de mousse are totally lacking up to now.

Therefore, the present study aimed to evaluate the effect of chitosan during the second fermentation and riddling stage of sparkling wines and to study the influence on sensory, foam and quality parameters of the finished product.

## 2. Materials and Methods

### 2.1. Chemicals

HPLC-grace acetonitrile, acetic acid, and methanol were obtained from Merck (Darmstadt, Germany). Milli-Q quality water was used for all HPLC experiments. Pure standards of volatile compounds, internal standard (2-octanol) and potassium metabisulphite were purchased from Sigma-Aldrich (Milano, Italy). Dichloromethane and methanol (SupraSolv) were supplied by Merck (Darmstadt, Germany), absolute ethanol (ACS grade) was obtained from Scharlau Chemie (Sentmenat, Spain) and pure water was obtained from a Milli-Q purification system (Millipore, Billerica, MA, USA). LiChrolut EN resin for solid-phase extraction (SPE) prepacked in 200 mg cartridges (3 mL total volume) were purchased from Merck (Darmstadt, Germany). Chitosan from *Aspergillus niger* (80–90% deacetylated, with average molecular weight of 10–30 kDa) was obtained from KitoZyme (Herstal, Belgium). 

### 2.2. Samples Preparation 

Base wines (75 mg/L total SO_2_), obtained from cv Pinot gris and Pignoletto grapes, were filtered under nitrogen and 25 g/L of beet sucrose was added and arranged in two distinct trials, consisting of 50 bottles each, the first with no further additions (CTRL) and a second with addition of fungoid chitosan (250 mg/L) (KT). Before closing with bidules and crown caps, samples were inoculated with rehydrated active dried yeasts (3 × 10^6^ cells of *Saccharomyces cerevisiae* strain 1042 from University of Bologna—ESAVE collection) and ammonium phosphate (200 mg/L) was added. Six bottles (three each trial) were provided with manometer to monitor the internal pressure development. All the bottles were left at controlled temperature (18 °C) during the prise de mousse that lasted about 1 month during which the pressure increase was annotated daily, and the bottles were agitated to facilitate the chitosan resuspension. Samples were analysed as base wines, at the end of secondary fermentation (1 month) and after 12 months of sur lie maturation (degorgement).

### 2.3. Oenological Parameters

All the analyses were carried out according to OIV methods [17]. The pH was determined by using a pH meter (Mettler Toledo, Columbus, OH, USA). The alcoholic strength of wines was determined with an oenochemical distilling unit (Gibertini, Italy). Total phenolics (TPI) were spectrophotometrically determined (after wine filtration at 0.45 μm with cellulose filters) at 280 nm using an Uvidec 610 spectrophotometer (Jasco, Tokyo, Japan), and results were expressed as mg/L of gallic acid (GAE). All the analyses were carried out in triplicate. The browning development of the wines was followed measuring the absorbance at 420 nm (1 cm optical path) after filtration (0.45 μm, cellulose filters) at each sampling time.

### 2.4. Organic Acids

Quantification of organic acids, sugars and glycerol was conducted following the procedure described by Chinnici et al. [18]. The HPLC used was a Jasco apparatus (Tokyo, Japan) equipped with a binary pump (PU 1580), a 20 μL loop, a Rheodyne valve (Cotati, CA, USA), a photodiode detector (PU MD 910; Tokyo, Japan) and a column oven (Hengoed, Mid Glamorgan, UK). The column was a Bio-Rad Aminex HPX 87H (300 mm × 7.8 mm), thermostated at 35 °C. Isocratic elution was carried out with 0.005 N phosphoric acid at flow 0.4 mL/min. All the analyses were carried out in triplicate. Organic acids were quantified using external calibration curves obtained with standard compounds at known concentrations.

### 2.5. Phenolic Acids

Phenolic acid analysis was performed following a previous method after minor modifications [19]. A Jasco HPLC instrument (Tokyo, Japan), equipped with a quaternary gradient pump Jasco PU-2089, an autosampler Jasco AS-2057 Plus Intelligent Sampler and two detectors, a Jasco UV/Vis MD-910 PDA detector and a Jasco FP-2020 Plus Fluorescence detector, was used. The column was a C18 Poroshell 120 (Agilent technologies, Santa Clara, CA, USA), 2.7 μm, (4.6 mm × 150 mm), operating at 35 °C with a flow of 0.8 mL/min. Elution solvents were 2% acetic acid in HPLC grade water (Eluent A) and 2% acetic acid in HPLC grade acetonitrile (Eluent B). Gradient elution was as follow: from 98% to 95% A in 10 min, 95% to 90% A in 7 min, 90 to 82% A in 6 min, 82% to 80% A in 3 min, 80% to 70% A in 3 min, 70% to 50% A in 3 min, 50% to 0% A in 4 min and 98% A in 1 min. Quantification of phenolic compounds was carried out using an external calibration curve obtained by injecting solutions of standard compounds at known concentrations and plotting peak areas vs. concentrations. The amount of tartrate esters of caffeic, coumaric and ferulic acids and Grape reaction Product GRP were expressed as the respective hydroxycinnamic acid.

### 2.6. Total Protein Content

A protein assay kit TP0300 from Merck (Darmstadt, Germany) was used to quantify soluble proteins of sparkling wines. The procedure described is based on Peterson’s modification of micro-Lowry method where known interferents (amino acid, peptide buffers and sucrose) were eliminated after protein precipitation with deoxycholate. Prior to analysis, wine samples were properly degasified and diluted 10 times with distilled water. Total protein concentrations are expressed in mg/L of BSA (bovine serum albumin).

### 2.7. Amino Acids and Amines

#### 2.7.1. Derivatization

A methodology proposed by Cejudo-Bastante et al. [20] was used. Briefly, 1.75 mL of borate buffer 1 M, 0.75 mL of methanol, 1 mL of sample and 20 μL of diethyl ethoxy methyl malonate (DEEMM) were left to react in a 10 mL screw-cap tube for 30 min in an ultrasound bath. Afterward, solution was warmed at 70 °C for 20 min in order to eliminate the excess of DEEMM. Once cooled, the samples were filtered with a 0.45 μm cellulose filter. 

#### 2.7.2. HPLC Analysis

HPLC separation was performed on the instrument already cited in Section 2.5. A Waters (Milford, MA, USA) reversed-phase column Nova-Pak^®^ C18 (3.9 mm × 300 mm; 4 μm), thermostated at 40 °C, was used. Mobile phases were A (25 mM acetate buffer pH = 5.65) and B (80:20 mixture of acetonitrile and methanol). Flow rate: 1.1 mL/min. HPLC gradient, for solvent A was: 0 min, 100%; 7 min, 96%; 18 min, 94%; 23 min, 92%; 25 min, 92%; 28 min, 85%; 50 min, 77%; 60 min, 55%; 65 min, 40%; 67 min, 20% and 70 min, 100%. 

Detection was performed at 280 nm while quantification was based upon calibration curves obtained by plotting peak areas vs. concentration of solutions of standard amino acids and amines at known concentration.

### 2.8. Determination of Mannose

The content of mannoproteins (expressed as mg/L of mannose) was determined in wines after 12 months of ageing on yeast lees. A 10 mL of wine was first concentrated up to 2 mL under vacuum and then precipitated using cooled ethanol and HCl following the method of Segarra et al. [21]. After acid hydrolysis [22], samples were analysed with the HPLC apparatus cited in Section 2.4 equipped with a refraction index detector (Jasco 830-RI; Tokyo, Japan). The column was a Transgenomic CarboSep CHO-682 (300 mm × 7.8 mm) set at 80 °C. Elution was carried out using deionized water with a flow rate of 0.4 mL/min. Quantification of mannose was performed by means of a calibration curve of standard solutions of known concentrations. 

### 2.9. Foamability

Analysis of foam quality was carried out by following a modified Mosalux method [23]. The instrument consisted of a glass column (400 mm × 24 mm), containing 50 mL of degasified wine to examine, with a porous septum (101–106 μm) at the base, which keeps the carbon dioxide separate from the wine, and a tap, necessary to block the flow of gas. A carbon dioxide cylinder was connected to the column, regulated at 1 bar and at a flow rate of 110 mL/min. Once the gas was opened, the evolution of the foam was recorded for 15 min. During this period, the height of the foam has been measured every 15 s. After 15 min, the cylinder and the column tap were closed, and the time required for the foam to disappear was measured. Three different parameters were measured: (i) HM, the maximum height reached by the foam after CO_2_ injection, expressed in mm, (ii) HS, the foam height stability during 15 min of CO_2_ injection, expressed in mm and (iii) TS, which is the foam stability time, expressed in seconds, once flow of CO_2_ is interrupted. 

### 2.10. Sensory Analysis 

Sensory analysis was performed by 14 (8 men and 6 women aged from 27 to 64) well-experienced panelists recruited from the staff of the Department of Agricultural and Food Sciences, trained according to ISO 8586:2012. Wines sensory attributes were set based on testing cards already established by our research group for sparkling wines and further developed by asking the panellists to assess samples for appearance (foam in particular), aroma, flavour, mouthfeel and aftertaste. A total of 10 attributes were selected by consensus including 3 for the appearance, 3 for the aroma and 4 for the mouthfeel/tactile. A Quantitative Descriptive Analysis (QDA) test was performed on a continuous unstructured scale left anchored from absent to maximum. All sessions were performed in normalized room according to ISO 8589:2007. Wine samples were first individually served in the presence of each panellist to evaluate the foaming characteristics. In a second session, each of the wines were poured immediately before being served to perform the aroma and mouthfeel assessment. Coded and capped wines glasses and white trays were used (ISO 3591:1977). Data were elaborated by means of analysis of variance (ANOVA) and Friedman test to evaluate sample, panelists and replication variability of data.

### 2.11. Wine Volatile Compounds

A method already described and validated by Lopez et al. [24] was used for volatile extraction. A hundred microliter of a 2-octanol solution at 500 mg/L was added to 20 mL of degassed wine as internal standard and deposed on a previously activated LiChrolut EN cartridge. Analytes were eluted with 5 mL of dichloromethane and concentrated to 200 μL under a stream of nitrogen prior to GC-MS analysis. The Trace GC ultra-apparatus coupled with a Trace DSQ mass selective detector (Thermo Fisher Scientific, Milan, Italy) was equipped with a fused silica capillary column Stabilwax-DA (Restek, Bellefonte, PA, USA; 30 m, 0.25 mm i.d. and 0.25 μm film thickness). The carrier gas was He at a constant flow of 1.0 mL/min. The GC programmed temperature was 45 °C (held for 3 min) to 100 °C (held for 1 min) at 3 °C/min and then to 240 °C (held for 10 min) at 5 °C/min. Splitless mode injection (1 μL) was performed at 250 °C. Detection was carried out by electron ionization (EI) mass spectrometry in full scan mode, using ionization energy of 70 eV. Transfer line interface was set at 220 °C and ion source at 260 °C. Mass acquisition range was m/z 30–400. Compounds were identified by a triple criterion: (i) by comparing their mass spectra and retention time with those of authentic standards, (ii) compounds lacking of standards were identified after matching their respective mass spectra with those present in the commercial libraries NIST 08 and Wiley 7 and (iii) matching the linear retention index (LRI) obtained under our conditions, with already published LRI on comparable polar columns. Quantification of compounds was carried out via the respective total ion current peak areas after normalization with the area of the internal standard. Calibration curves were obtained by injections of standard solutions, subjected to the already cited extraction procedure, containing a mixture of commercial standard compounds at concentrations between 0.01 and 200 mg/L, and internal standard at the same concentration as in the samples. The calibration equations for each compound were obtained by plotting the peak area response ratio (target compound/internal standard) versus the corresponding concentration. For compounds lacking reference standards, the calibration curves of standards with similar chemical structure were used. Analyses were done in triplicate. 

### 2.12. Statistical Analysis

Physicochemical data were given as mean ± SD. Evaluation of statistical significance was conducted by one-way analysis of variance (ANOVA) followed by a post hoc comparison Tukey test. Differences between groups were considered significant when *p* < 0.05. The univariate analysis (ANOVA) was performed using XLSTAT version 2016.02 (Addinsoft, Paris, France).

## 3. Results

### 3.1. Oenological Parameters

No significant differences between the treatments were recorded for the main oenological parameters (Table 1). Volatile acidity, pH and alcohol strength were adequate for this type of product. Yellow colour was subjected to little variations during the fining period, regardless the treatment adopted. After the end of the secondary fermentation, both samples showed a tendency to marginally increase the titratable acidity. This was followed by a subsequent reduction during the 12 months of ageing in the presence of yeast lees. This last evidence will be further discussed in the following section.

### 3.2. Organic Acids and Glycerol

HPLC quantification of organic acids after secondary fermentation showed similar values for both CTRL and KT samples (Table 2). As already reported by Pozo-Bayon et al. [25], glycerol content tended to slightly augment (by 0.2 g/L in our samples) after second fermentation because of yeast production, remaining unchanged for the following storage period. After 12 months of on lees ageing, concentration of tartaric acid was significantly decreased in both samples, which contributed to the reduction of titratable acidity reported in the previous section. The concentration of pyruvic acid, a secondary metabolite of alcoholic fermentation, increased in both samples after 12 months of ageing “sur lie” indicating its release from yeast cells autolysis. 

### 3.3. Protein Content

If compared with base wine, after secondary fermentation total proteins increased in both CTRL and KT samples to the same extent (Table 1). This was somehow expected since, as already reported, yeast metabolism and initial autolysis favour the release of proteins and peptides from cell cytoplasm to the wine since the very beginning of the ageing [5].

However, at 12 months, protein content further increased in KT sparkling wines while in CTRL samples, a decrease was observed. Untreated samples followed the common pattern already observed by Nunez and coworkers [5] where late reduction of protein content during “sur lies” ageing could be attributed to both the residual cells protease activity and the presence of alcohol [26,27].

Nevertheless, in KT samples, interactions between positively charged amine groups of the polymer and negatively charged components of cell wall may occur [14,28], which promotes an increased cell permeability, further speeding up the process of yeast autolysis and the release of proteins.

### 3.4. Phenolic Acids

Evolution of phenolic compounds after secondary alcoholic fermentation and 12 months of ageing on lees is presented in Table 3. A total of 18 compounds were identified in both the sparkling wines. Generally, treatments with chitosan did not affect the polyphenolic profile of wines compared to control samples, with the exception of (+)-catechin, which was present in significantly lower amounts (*p* < 0.05) after 12 months of storage in the presence of the biopolymer. This is due to the affinity of chitosan for flavanols present in wines, leading to its absorptive removal [16,29]. Overall, after 12 months of permanence on yeast lees, phenols slightly diminished or, in some cases, remained unchanged with respect to the base wine. As already evidenced elsewhere [25,30], at reducing conditions like those of sparkling wines, phenolic acids concentration tends not to be considerably modified, because of the scarcity of dissolved oxygen and the protective role of CO_2_ against phenolic oxidation. Table 3 also evidences a temporary diminution of almost all the phenolic compounds just after the secondary fermentation. This has been often observed, due to absorption of phenolics onto yeast cells [25,31]. During the subsequent period of lees ageing, two concurrent phenomena are then expected to be occurred: (i) the partial release of those phenols into the wine, following the cell disorganization and (ii) the hydrolysis of hydroxycinnamates esters that promotes the increase of the corresponding phenolic acids [32].

### 3.5. Amino Acids and Amines

The data relative to amino acids (Table 4) illustrate the typical decrease in their total amount following the second fermentation because of the assimilation by yeasts [26,33]. By comparing the concentrations in base and refermented wines it appears, in fact, that apart from asparagine and glutamine, all the amino acids where metabolized by yeasts to various extent. It should, however, be considered that at the end of fermentation, residual nitrogen composition of wines depends on a balance between initial depletion by yeasts and successive excretion or passive exsorption, these occurring latter during the last phases of fermentation [23,29]. In addition, it is worth noting that when compared to untreated wines (CTRL), KT seemed to elicit a generalized lower consumption (or higher excretion) of amino acids, particularly with respect to glycine, arginine and lysine, that drove to significantly higher final amounts of amino acids for chitosan treated wines, at the end of secondary fermentation. After ageing on lees, amino acids significantly increased (Table 4). This evidence is in accordance with that obtained in previous works [33,34] where the cellular pool of amino acids has been claimed to be released to the medium by exsorption after yeast cell degradation. During the permanence on lees, both the treated and untreated samples evolved in a very similar way, maintaining the differences already recorded after the second fermentation, being the KT samples richer in these compounds with respect to CTRL. For what concern amines, their total amount did not change noticeably during the distinct production phases (Table 4).

Individual changes were found for putrescine which diminished after secondary fermentation in all the samples, partially counterbalanced by little and progressive increase in ornithine amounts independently of the treatments.

### 3.6. Foamability Parameters

**Figure 1:**
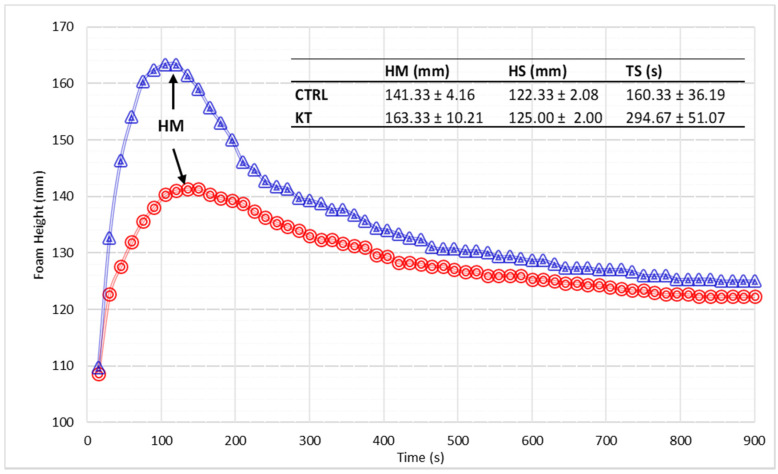
Evolution of the foam height during 15 min of measurement of wine sampled after 12 months of “sur lie” ageing. In the inset are outlined the recorded foam parameters HM = maximum foam height; HS = stability height; TS = stability time. Control (CTRL) (**-O-**) Chitosan (KT) (**-∆-**).

A notable portion of the perceived quality of sparkling wines is linked to foam features. For this reason, foamability was analysed on samples after 12 months of ageing. Foam profile and related parameters are reported in Figure 1. Results show higher values for foam height (HM) and stability time (TS) in KT samples when compared to CTRL. This could be correlated to the higher content of proteins in wines aged in the presence of the polysaccharide (Table 1) as already commented above. The pivotal role of proteins on foam quality has been studied by several researchers [5,35,36].

Those authors demonstrated that released proteins from yeast cell autolysis would improve foam development and stability in wines by reducing surface tension and increasing viscosity. Furthermore, in addition to proteins, amino acids have also been considered as foaming agents [37]. Their action is associated with the positive charge that these molecules carry in acidic wine conditions, resulting in the presence of both hydrophilic and hydrophobic groups. As with proteins, this favours the retention of amino acids in the air–liquid interphase, improving wine foamability [38]. Amines have been found to behave in a similar way [37].

Mannoproteins are another wine component consistently reported to positively affect foam height and stability [37]. In our samples, however, after 12 months of ageing on lees, we did not find significant differences in mannose content of wines (114 and 124 mg/L for KT and CTRL, respectively), suggesting that such polysaccharides could not be the reason for the better foam quality in chitosan-treated sparkling samples.

### 3.7. Evolution of Volatile Compounds during Traditional Sparkling Winemaking Process

**Figure 2:**
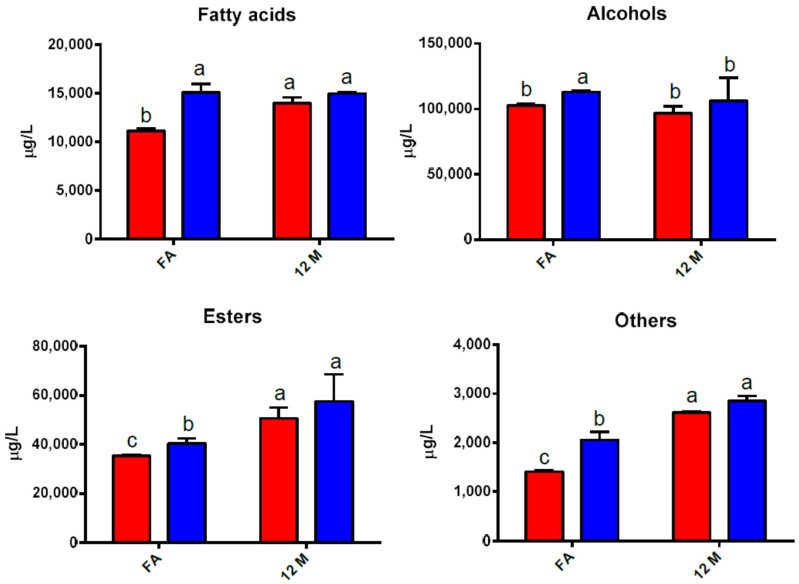
Concentrations (μg/L) of volatile compounds grouped by chemical family after secondary alcoholic fermentation (2nd AF) and after 12 months of storage “sur lie.” Different letters indicate significant differences according to Tukey’s test (*p* < 0.05). *n* = 3.

The most significant volatile compounds identified in sparkling wines after the secondary fermentation and after 12 months of maturation on yeasts lees are reported in Table 5. Figure 2 also shows the sum of volatile compounds grouped by chemical families in order to be separately discussed.

#### 3.7.1. Fatty Acids

Our results suggested that the presence of chitosan during the secondary fermentation generally enhanced the release of volatile fatty acids (Figure 2), likely impacting the aromatic profile of wines [41]. Similar results were reported in a previous work where chitosan was added in white musts during alcoholic fermentation [14]. Fatty acids are important constituents of cell membranes. Electrostatic interactions between chitosan amine groups and negatively charged cell surface components may induce an increase of permeability of yeast cell membranes, energetic unbalance and augmented excretion of fatty acids synthesised inside the cell [28,42,43]. Regarding the ageing period, a slight rising of some fatty acids was observed in both samples, with amounts of medium chain fatty acids such as 3-hydroxybutanoic, hexanoic, octanoic and decanoic increasing with time (Figure 2 and Table S1). This trend that could lead to an impact on the sensory attributes of final sparkling wines will be further discussed in a following section. 

#### 3.7.2. Alcohols

Alcohols are related to an intense odour and play and important role in wine aroma. At concentrations lower than 300 mg/L, for instance, higher alcohols can impart wine complexity, but, at higher amounts, their intense odour could harm wine finesse [44]. None of our samples exceed the critical threshold (Figure 2), all reaching concentration levels around 100 mg/L as a sum.

Interestingly, after the second fermentation, the formation of volatile alcohols seemed to be slightly, though not significantly, higher in KT samples (10 mg/L higher as a sum). Some of these compounds are synthesised by yeast metabolism of sugars or amino acids by means of the Ehrlich pathway [45]. Isobutanol, in particular, was found at higher amounts after second fermentation in the presence of chitosan (Table S1). This alcohol comes from valine degradation by *Saccharomyces cerevisiae*, via the sequential formation of a α-ketoacid (ketoisovalerate), which is then reduced to isovaleraldehyde [46]. This latter can either be reduced to isobutyl alcohol or oxidized to isobutyric acid, which, also, was found at higher amounts in KT wines (Table S1). The reason of this metabolic expression in the presence of chitosan remains unclear. 

Total amount of alcohols substantially did not change after 12 months of ageing on lees, but changes did occur for some compounds, independently from the sample considered.

A major variation in content was found for 2-phenylethanol, which at the end of ageing was reduced by about 10 mg/L with respect the initial amount (Table S1). This would impact the sensory features of the wines, considering the rose-reminiscent note of this alcohol.

#### 3.7.3. Esters

The presence and evolution of volatile esters in winemaking is of great interest since they play a fundamental role in the sensory properties of wines, imparting pleasant aromatic character such as candy, perfume-like and fruitness flavour [47]. Evolution of volatile esters in both samples during traditional sparkling winemaking is shown in Figure 2. Again, generation of these compounds was favoured by the presence of chitosan when compared to the control samples. However, this evidence was only significative at the end of secondary fermentation and seemed not to be further present after on-lees ageing.

Esters are generated from the reaction between alcohols and acids [45]. Therefore, an enhancement of the esterification reaction due to the greater availability of some volatile alcohols and fatty acids on KT wines after secondary fermentation (see Section 3.7.2) could be the origin of the increased content of esters in samples treated with chitosan. For example, isoamyl acetate, one of the most important acetate esters in wines, known for its distinctive banana aroma, was produced in higher concentrations in KT samples after secondary fermentation with *S. cerevisiae* (Table S1). As expected, some esters (acetates in particular) tended to decrease with time, with notable exceptions for the ethyl esters of some carboxylic acids (succinic, tartaric and lactic), which are usually regarded as markers of aged sparkling wines [4] and altogether contribute to the overall increase of this chemical class after 12 months of ageing (Figure 2). 

#### 3.7.4. Other Compounds

The combined sum of some compounds, such as heterocyclic dioxane and dioxolane (generated from the acetylation between acetaldehyde and glycerol) or furans and pyrazines produced after the Maillard reaction between monosaccharides and amino acids, is also shown in Figure 2. This graph also comprises some carbonyl compounds (ketones and aldehydes) included in Table S1 under the common name of “others.” As displayed in Figure 2, the presence of chitosan generally led to higher levels of these compounds, especially just after the secondary fermentation. Specifically, the major contributors to this higher level on KT samples after second fermentation are acetoin, 2-hydroxy-3-pentanone, ethyl-5-oxotetrahydro-2-furancarboxylate and 2,3-dihydroxypyrazine (Table S1). These compounds may contribute to pleasant, buttery and nutty nuances.

Further, after the ageing period, an overall increase of these compounds was observed, where samples treated with chitosan continue to show greater richness in these volatile compounds, mainly due to the presence of acetovanillone and 2,3-dihydroxypyrazine. 

### 3.8. Sensory Profile of Sparkling Wines after Secondary Fermentation and after 12 Months of Ageing Sur Lie

**Figure 3:**
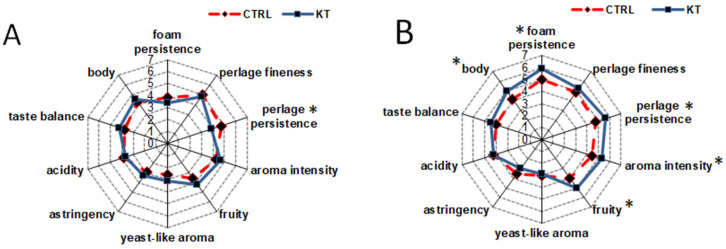
Sensory evaluation of sparkling wines after secondary fermentation (**A**) and 12 months of ageing “sur lie” (**B**). * Indicates significant differences according to Tukey’s test (*p* < 0.05).

Sensory analysis was carried out after fermentation and after 12 months of ageing “sur lie” (Figure 3). As depicted on Figure 3A, no significant differences were appreciated at the end of secondary fermentation except for perlage persistence, which was higher in CTRL wines.

However, after 12 months of ageing in the presence of lees, the judges did find differences in the aromatic profile and foamability. Regarding the former, the richness in volatile compounds after ageing period (See Section 3.7) seemed to determine some impact to the wines, and KT samples were judged as the ones with higher aromatic intensity and richer fruity character. Despite the lack of significant differences between the distinct classes of volatiles of aged wines, in fact, the overall higher contents of aromatic compounds, especially some acids and esters (Table S1), has certainly contributed to this result. Sensory analysis also confirmed the data reported on Section 3.6 regarding foaming properties, as both perlage and foam persistence were significantly higher in wines added with chitosan because of the enhanced content of proteins and amino acids. Treated wines, in addition, were rated as more bodied and structured.

## 4. Conclusions

Based on our results, it was confirmed that the use of chitosan in traditional sparkling wines production may result in a higher content of fixed (mainly proteins and amino acids) and volatile compounds. This evidence could be associated to the ability of chitosan to interact with both the wall and the membrane of yeasts cells by electrostatic interactions at wine pH. This would eventually lead to the increase of permeability and the augmentation in the release of the cited compounds. Furthermore, this trend had an impact on the overall quality of wines, by increasing foamability and aromatic profile, making chitosan an interesting tool for the production of sparkling wines.

## Figures and Tables

**Table 1:** Oenological parameters and total protein concentration of the samples after secondary alcoholic fermentation (2nd AF) and after 12 months of ageing “sur lie.” In the same row, different letters indicate significant differences according to Tukey’s test (*p* < 0.05). *n* = 3. TPI = total phenolics; GAE = gallic acid equivalent.

	Base Wine	2nd AF		12 Months “Sur lie”	
		CTRL	KT	CTRL	KT
Titratable acidity (g/L)	5.75 ± 0.07 ^ab^	5.90 ± 0.14 ^a^	5.85 ± 0.07 ^a^	5.59 ± 0.06 ^ab^	5.51 ± 0.01 ^b^
pH	3.10 ± 0.01 ^a^	3.10 ± 0.01 ^a^	3.10 ± 0.02 ^a^	3.11 ± 0.01 ^a^	3.12 ± 0.01 ^a^
Volatile acidity (g/L)	0.29 ± 0.02 ^a^	0.28 ± 0.01 ^a^	0.30 ± 0.01 ^a^	0.32 ± 0.01 ^a^	0.28 ± 0.01 ^a^
Alcohol (%*v*/*v*)	10.32 ± 0.71 ^b^	11.42 ± 0.09 ^a^	11.36 ± 0.19 ^a^	11.40 ± 0.06 ^a^	11.30 ± 0.03 ^a^
Optical Density 420 nm	0.092 ± 0.001 ^a^	0.093 ± 0.01 ^a^	0.089 ± 0.012 ^a^	0.101 ± 0.001 ^a^	0.104 ± 0.001 ^a^
TPI (GAE)	172.3 ± 0.02 ^ab^	162.8 ± 0.07 ^b^	180.4 ± 0.28 ^a^	177.1 ± 0.03 ^ab^	176.9 ± 0.03 ^ab^
Total proteins (mg/L)	22.43 ± 0.45 ^d^	30.53 ± 1.28 ^b^	32.99 ± 1.37 ^b^	25.55 ± 0.85 ^c^	38.25 ± 1.01 ^a^

**Table 2:** Organic acids and glycerol amounts (g/L) after secondary alcoholic fermentation (2nd AF) and after 12 months of storage “sur lie” (shikimic and pyruvic acids as mg/L). In the same row, different letters indicate significant differences according to Tukey’s test (*p* < 0.05). *n* = 3.

	Base Wine	2nd AF		12 Months “Sur lie”	
		CTRL	KT	CTRL	KT
Tartaric acid	3.57 ± 0.01 ^a^	3.58 ± 0.14 ^a^	3.66 ± 0.04 ^a^	2.53 ± 0.02 ^b^	2.61 ± 0.14 ^b^
Pyruvic acid	26.1 ± 0.23 ^b^	24.3 ± 2.19 ^b^	22.4 ± 1.46 ^b^	36.8 ± 0.42 ^a^	40.6 ± 1.54 ^a^
Malic acid	0.13 ± 0.01 ^a^	0.16 ± 0.04 ^a^	0.14 ± 0.01 ^a^	0.19 ± 0.01 ^a^	0.19 ± 0.01 ^a^
Shikimic acid	60.7 ± 0.35 ^a^	54.4 ± 2.15 ^a^	56.1 ± 0.75 ^a^	56.3 ± 1.22 ^a^	55.9 ± 1.22 ^a^
Lactic acid	2.37 ± 0.03 ^a^	2.30 ± 0.08 ^a^	2.35 ± 0.07 ^a^	2.35 ± 0.08 ^a^	2.36 ± 0.08 ^a^
Acetic acid	0.18 ± 0.01 ^ab^	0.16 ± 0.01 ^bc^	0.19 ± 0.02 ^a^	0.13 ± 0.01 ^d^	0.14 ± 0.01 ^cd^
Succinic acid	0.55 ± 0.01 ^a^	0.52 ± 0.04 ^a^	0.60 ± 0.03 ^a^	0.69 ± 0.06 ^a^	0.69 ± 0.06 ^a^
Glycerol	3.21 ± 0.05 ^a^	3.39 ± 0.12 ^a^	3.40 ± 0.11 ^a^	3.40 ± 0.12 ^a^	3.35 ± 0.10 ^a^
Sum	6.80 ± 0.03 ^a^	6.70 ± 0.22 ^a^	6.93 ± 0.08 ^a^	5.88 ± 0.16 ^b^	5.98 ± 0.16 ^b^

**Table 3:** Concentrations (mg/L) of phenolic acids after secondary alcoholic fermentation (2nd AF) and after 12 months of storage “sur lie.” In the same row, different letters indicate significant differences according to Tukey’s test (*p* < 0.05). *n* = 3. GRP = Grape reaction Product.

	Base Wine	2nd AF		12 Months “Sur lie”	
		CTRL	KT	CTRL	KT
*Hydroxybenzoic acids and flavanols*					
Gallic	21.79 ± 0.26 ^a^	21.21 ± 0.50 ^a^	21.05 ± 1.04 ^a^	23.17 ± 0.16 ^a^	22.69 ± 0.07 ^a^
Syringic	0.74 ± 0.05 ^a^	0.85 ± 0.07 ^a^	1.04 ± 0.03 ^a^	1.18 ± 0.03 ^a^	0.94 ± 0.49 ^a^
*p*-Hydroxybenzoic	1.15 ± 0.01 ^a^	0.15 ± 0.02 ^c^	0.09 ± 0.09 ^c^	0.77 ± 0.22 ^b^	0.62 ± 0.04 ^b^
(+)-Catechin	3.58 ± 0.07 ^a^	3.53 ± 0.12 ^a^	3.54 ± 0.21 ^a^	3.16 ± 0.03 ^a^	2.60 ± 0.04 ^b^
*Hydroxycinnamic acids*					
*t*-Caftaric acid	5.39 ± 0.08 ^a^	4.14 ± 0.05 ^c^	4.12 ± 0.08 ^c^	4.64 ± 0.01 ^b^	4.49 ± 0.03 ^bc^
GRP	5.81 ± 0.09 ^a^	3.47 ± 0.10 ^b^	3.17 ± 0.05 ^c^	5.87 ± 0.03 ^a^	5.75 ± 0.06 ^a^
*t*-Coutaric acid	1.92 ± 0.06 ^a^	1.87 ± 0.02 ^a^	1.87 ± 0.01 ^a^	1.89 ± 0.08 ^a^	1.83 ± 0.01 ^a^
*c*-Coutaric acid	2.46 ± 0.01 ^a^	1.37 ± 0.01 ^bc^	1.36 ± 0.01 ^c^	1.69 ± 0.05 ^b^	1.63 ± 0.02 ^b^
Fertaric acid	4.13 ± 0.07 ^a^	3.11 ± 0.02 ^c^	3.07 ± 0.06 ^c^	3.60 ± 0.01 ^b^	3.65 ± 0.07 ^b^
Caffeic acid	1.72 ± 0.01 ^a^	0.81 ± 0.11 ^bc^	0.78 ± 0.16 ^c^	1.16 ± 0.07 ^b^	1.11 ± 0.03 ^b^
*p*-Coumaric acid	1.57 ± 0.05 ^a^	0.56 ± 0.10 ^c^	0.51 ± 0.04 ^c^	0.89 ± 0.01 ^b^	0.92 ± 0.04 ^b^
Ferulic acid	1.74 ± 0.02 ^a^	0.77 ± 0.08 ^c^	0.76 ± 0.05 ^c^	0.98 ± 0.05 ^b^	0.97 ± 0.04 ^b^
*Flavonols*					
Quercetin	0.11 ± 0.01 ^a^	0.12 ± 0.01 ^a^	0.09 ± 0.01 ^a^	0.11 ± 0.01 ^a^	0.10 ± 0.01 ^a^
*Other*					
Tyrosol	3.20 ± 0.2 ^b^	3.81 ± 0.04 ^a^	3.72 ± 0.13 ^a^	3.94 ± 0.05 ^a^	3.81 ± 0.05 ^a^

**Table 4:** Concentrations (mg/L ± STD) of amino acids, ammonium ion and amines after secondary alcoholic fermentation (2nd AF) and after 12 months of storage “sur lie.” In the same row, different letters indicate significant differences according to Tukey’s test (*p* < 0.05). *n* = 3.

	Base Wine	2nd AF		12 Months Storage	
		CTRL	KT	CTRL	KT
Aspartic acid	7.45 ± 0.02 ^a^	1.34 ± 0.03 ^e^	1.67 ± 0.04 ^d^	3.29 ± 0.05 ^c^	3.65 ± 0.03 ^b^
Glutamic acid	10.52 ± 0.88 ^a^	4.24 ± 0.20 ^d^	5.82 ± 0.00 ^cd^	6.40 ± 0.05 ^bc^	8.01 ± 0.09 ^b^
Serine	6.41 ± 0.56 ^a^	0.97 ± 0.03 ^b^	1.02 ± 0.03 ^b^	1.95 ± 0.01 ^b^	1.94 ± 0.04 ^b^
Asparagine	4.70 ± 0.23 ^a^	5.27 ± 0.00 ^a^	5.11 ± 0.17 ^a^	5.56 ± 0.52 ^a^	5.71 ± 0.14 ^a^
Glutamine	25.65 ± 1.72 ^b^	34.38 ± 0.44 ^a^	34.27 ± 0.75 ^a^	36.34 ± 0.79 ^a^	34.31 ± 1.76 ^a^
Glycine	55.04 ± 2.17 ^a^	43.37 ± 0.05 ^c^	49.08 ± 1.04 ^b^	51.89 ± 0.25 ^ab^	56.97 ± 1.77 ^a^
Histidine	17.57 ± 0.19 ^a^	7.55 ± 0.10 ^c^	7.95 ± 0.19 ^c^	10.10 ± 0.12 ^b^	10.58 ± 0.23 ^b^
Threonine	2.20 ± 0.76 ^a^	1.00 ± 0.06 ^a^	0.91 ± 0.05 ^a^	1.47 ± 0.06 ^a^	1.55 ± 0.08 ^a^
Arginine	17.94 ± 0.04 ^b^	14.43 ± 0.09 ^d^	16.47 ± 0.22 ^c^	16.45 ± 0.08 ^c^	19.23 ± 0.28 ^a^
Alanine	6.52 ± 0.09 ^a^	2.23 ± 0.07 ^c^	3.03 ± 0.11 ^bc^	3.23 ± 0.30 ^b^	3.42 ± 0.30 ^b^
Tyrosine	4.84 ± 0.03 ^b^	3.71 ± 0.01 ^d^	4.18 ± 0.07 ^c^	4.80 ± 0.00 ^b^	5.21 ± 0.01 ^a^
Ammonium	28.43 ± 0.43 ^c^	45.98 ± 0.51 ^b^	45.61 ± 0.67 ^b^	47.34 ± 0.59 ^ab^	48.56 ± 0.20 ^a^
Ethanolamine	15.36 ± 0.12 ^c^	15.43 ± 0.20 ^c^	15.80 ± 0.07 ^bc^	16.4 ± 0.23 ^ab^	16.45 ± 0.08 ^a^
Valine	10.43 ± 0.89 ^a^	3.32 ± 0.04 ^c^	4.62 ± 0.06 ^bc^	4.79 ± 0.03 ^bc^	6.01 ± 0.06 ^b^
Methionine	5.59 ± 0.07 ^a^	1.85 ± 0.15 ^b^	1.93 ± 0.20 ^b^	1.90 ± 0.18 ^b^	1.98 ± 0.26 ^b^
Isoleucine	9.61 ± 0.69 ^a^	0.94 ± 0.00 ^c^	1.30 ± 0.16 ^c^	2.14 ± 0.17 ^bc^	2.65 ± 0.02 ^b^
Leucine	18.64 ± 0.32 ^a^	3.94 ± 0.03 ^e^	4.92 ± 0.12 ^d^	5.87 ± 0.02 ^c^	6.83 ± 0.05 ^b^
Phenylalanine	7.10 ± 0.08 ^a^	2.57 ± 0.07 ^d^	3.45 ± 0.07 ^c^	3.34 ± 0.02 ^c^	4.38 ± 0.15 ^b^
Ornithine	2.46 ± 0.14 ^d^	4.05 ± 0.06 ^bc^	3.90 ± 0.06 ^c^	4.52 ± 0.04 ^a^	4.36 ± 0.13 ^ab^
Lysine	35.61 ± 0.27 ^a^	11.38 ± 0.60 ^d^	14.29 ± 1.14 ^c^	15.63 ± 0.05 ^c^	18.6 ± 0.48 ^b^
Putrescine	18.93 ± 0.42 ^a^	14.48 ± 0.84 ^b^	15.07 ± 0.44 ^b^	15.38 ± 0.44 ^b^	16.38 ± 0.58 ^b^
SUM amino acids	248.3 ± 3.46 ^a^	146.5 ± 0.32 ^e^	163.9 ± 3.04 ^d^	179.7 ± 1.94 ^c^	195.4 ± 4.31 ^b^
SUM amines	36.75 ± 0.15 ^a^	34.96 ± 1.43 ^a^	35.77 ± 1.58 ^a^	36.3 ± 0.39 ^a^	37.19 ± 0.10 ^a^

**Table 5:** List of identified compounds, HMF = 5-hydroxymethylfurfural. ^a^ Identification assignment: Std = comparing mass spectra, linear retention index (LRI) and retention times with pure compounds, MS = by comparing mass spectra with NIST 08 and Wiley 7 spectral database, LRI = matching LRI on comparable polar columns (taken from the following publicly available databases: [39,40]).

Compound	tR (min)	LRI	Identification ^a^
Isobutyl alcohol	5.70	1106	Std, MS, LRI
Isoamyl acetate	6.74	1133	Std, MS, LRI
n-butanol	7.19	1145	Std, MS, LRI
3-penten-2-ol	7.80	1149	Std, MS, LRI
3-methyl-1-butanol	8.92	1190	Std, MS, LRI
Ethyl n-caproate	9.86	1218	Std, MS, LRI
Ethyl pyruvate	11.33	1267	Std, MS, LRI
2-hexanol	12.47	1304	MS, LRI
3-methyl-1-pentanol	13.51	1331	Std, MS, LRI
Ethyl lactate	13.86	1340	Std, MS, LRI
n-hexanol	14.19	1349	Std, MS, LRI
2-hydroxy-3-pentanone	14.63	1360	Std, MS, LRI
3-ethoxy-1-propanol	15.10	1372	Std, MS, LRI
3-hexen-1-ol	15.37	1379	Std, MS, LRI
Ethyl octanoate	17.40	1432	Std, MS, LRI
Linalool oxide	18.60	1463	SMS, LRI
Furfural	18.78	1467	Std, MS, LRI
*c*-5-hydroxy-2-methyl-1,3-dioxane	20.19	1503	MS, LRI
Ethyl-3-hydroxybutyrate	21.05	1524	Std, MS, LRI
2-methyl-3-thiolannone	21.36	1531	MS, LRI
2,3-butanediol	23.08	1572	Std, MS, LRI
Ethyl 3-hydroxypropionate	23.93	1584	MS, LRI
*t*-4-hydroxymethyl-2-methyl-1,3 dioxolane	24.35	1606	MS, LRI
2-furancarboxylic acid, ethyl ester	24.55	1616	MS, LRI
n-butyric acid	24.71	1624	Std, MS, LRI
Decanoic acid, ethyl ester	25.38	1659	Std, MS, LRI
Pentanoic acid	25.87	1689	MS, LRI
Furfuryl alcohol	26.02	1695	Std, MS, LRI
Diethyl succinate	26.44	1710	Std, MS, LRI
3-methylthio-1-propanol	27.48	1746	Std, MS, LRI
1,3-propanediol diacetate	28.03	1766	MS, LRI
Ethyl 4-hydroxybutanoate	29.79	1840	Std, MS, LRI
2-phenylethyl-acetate	30.01	1851	Std, MS, LRI
*t*-5-hydroxy-2-methyl-1,3-dioxane	30.11	1856	MS, LRI
Hexanoic acid	30.39	1870	Std, MS, LRI
Benzyl alcohol	31.16	1905	Std, MS, LRI
2-phenylethanol	31.92	1931	Std, MS, LRI
Benzothiazole	32.96	1966	MS, LRI
2,3-dihydroxypyrazine	33.99	2001	Std, MS, LRI
Diethyl Malate	34.70	2038	MS, LRI
Octanoic acid	34.96	2052	Std, MS, LRI
Diethyl-2-hydroxypentanedioate	37.32	2197	Std, MS, LRI
4-vinyl-2-methoxyphenol	37.78	2220	Std, MS, LRI
Ethyl 5-oxotetrahydrofuran-2-furancarboxylate	38.82	2270	MS, LRI
decanoic acid	39.31	2293	Std, MS, LRI
Ethyl 2-hydroxy-3-phenylpropanoate	39.39	2297	MS, LRI
Glycerol	40.20	2328	Std, MS, LRI
Diethyl tartrate	40.33	2182	MS, LRI
Ethyl hydrogen succinate	40.94	2356	MS, LRI
4-vinyl phenol	41.26	2368	Std, MS, LRI
Benzoic acid	41.85	2390	Std, MS, LRI
3-furoic acid	42.08	2399	MS, LRI
Dodecanoic acid	42.78	2444	Std, MS, LRI
HMF	43.12	2467	Std, MS, LRI
Acetovanillone	47.96	2662	MS, LRI
n-hexadecanoic acid	49.08	2803	Std, MS, LRI
4-hydroxy-benzenethanol	51.20	2917	Std, MS, LRI
Octadecanoic acid	53.25	2998	Std, MS, LRI

## Supplementary Materials

The following are available online at https://www.mdpi.com/2304-8158/9/9/1174/s1, Table S1: Volatile compounds (mg/L) detected after second alcoholic fermentation (2nd AF) and after 12 months of storage “sur lie” in control (CTRL) and chitosan treated (KT) samples. In the same row, different letters indicate significant differences according to Tukey’s test (*p* < 0.05). *n* = 3.
