# Supplementary material for: Chitosan in Sparkling Wines Produced by the Traditional Method: Influence of Its Presence during the Secondary Fermentation
Source: Foods. 2020 Aug 25;9(9):1174. doi: 10.3390/foods9091174 (PMC7555991; doi:10.3390/foods9091174)
Supplement: Supplementary file 1 [file foods-09-01174-s001.pdf]

Table S1: Volatile compounds (mg/L) detected after second alcoholic fermentation (2nd AF) and after 12 months of storage "sur lie" in control (CTRL) and chitosan treated (KT) samples. In the same row, different letters indicate significant differences according to Tukey's test ($p < 0.05$). $n = 3$.

Compound		Sample							
Acids		CTRL_2ndAF		KT 2ndAF		CTRL_12M		KT_12M	
Isobutyric acid		138.85	c	343.51	b	681.92	a	662.07	a
Butanoic acid		450.39	b	546.82	ab	693.80	a	693.88	a
Pentanoic acid		322.51	c	429.56	b	458.33	a	472.92	a
n-Hexanoic acid		3522.00	b	4628.71	a	4010.38	ab	4320.18	ab
Octanoic acid		5343.60	b	6849.02	a	5975.78	ab	6364.81	ab
Decanoic acid		897.58	b	1623.73	a	1032.51	b	1168.24	ab
Benzoic acid		n.d.		n.d.		49.12	a	53.60	a
3-Furancarboxylic acid		132.37	b	215.43	b	615.71	a	589.72	a
Dodecanoic acid		54.00	b	87.68	a	84.46	ab	85.29	ab
Myristic acid		34.49	a	32.30	a	n.d.		n.d.	
Palmitic acid		131.44	b	205.32	ab	219.57	ab	310.22	a
Octadecanoic acid		90.07	c	138.23	bc	151.68	b	273.03	a
	sum	11117.30	b	15100.30	a	13973.26	a	14993.94	a
Alcohols									
Isobutyl alcohol		1797.79	c	2696.20	b	3628.61	a	3194.76	ab
n-Butanol		21.58	b	28.24	b	45.41	a	40.25	a
3-Penten-2-ol		353.30	a	315.34	a	97.77	b	96.79	b
3-Methyl-1-butanol		64819.35	a	69251.64	a	60668.26	b	65410.32	a
2-Hexanol		327.29	a	265.77	b	41.21	c	49.64	c
3-Methyl-1-pentanol		19.16	c	24.23	bc	36.23	ab	43.23	a
n-Hexanol		394.22	a	431.55	a	414.87	a	435.65	a
3-ethoxy-1-propanol		22.48	a	21.70	a	27.69	a	26.48	a
3-Hexen-1-ol		349.88	a	385.37	a	397.35	a	384.58	a
(s)-3-ethyl-4-methyl-1-pentanol		12.21	b	22.46	b	63.96	a	55.16	a
Butane-2,3-diol		1379.79	b	1873.32	a	1918.33	a	1901.45	a

Compound		Sample							
1-Octanol		n.d.		n.d.		28.92	a	33.42	a
1-methoxy-2-butanol		20.37	b	18.36	b	58.01	a	62.44	a
Furfuryl alcohol		13.23	b	22.91	a	16.87	ab	20.71	ab
3-(methylthio) 1-Propanol		233.97	b	321.25	ab	418.04	a	392.86	a
1,3-Propanediol, diacetate		59.09	b	91.04	a	31.72	c	42.48	c
Benzyl Alcohol		174.32	b	224.59	ab	327.31	a	306.02	a
2-phenylethanol		26507.25	ab	29177.35	a	17895.51	b	21351.30	ab
4-vinyl-2-methoxyphenol		29.71	b	38.99	b	122.44	a	132.37	a
Glycerol		69.89	c	197.23	bc	675.86	a	397.91	ab
4-vinylphenol		91.93	b	145.26	b	310.59	a	321.00	a
4-hydroxy-benzeneethanol		5617.09	b	7036.45	ab	8834.09	a	11001.97	a
	sum	102313.90	b	112589.25	a	96059.05	b	105700.79	b
Esters									
Isoamyl acetate		827.96	b	1031.97	a	202.64	c	171.08	c
Ethyl caproate		742.17	a	774.61	a	757.80	a	702.23	a
Ethyl pyruvate		26.97	c	21.13	c	91.12	a	76.05	b
Ethyl lactate		12180.48	ab	11268.33	b	14401.08	ab	16290.43	a
Ethyl caprylate		1037.45	a	1059.89	a	841.35	b	792.66	b
Ethyl 3-hydroxypropionate		34.70	b	40.45	ab	40.92	ab	51.41	a
2-Furancarboxylic acid, ethyl ester		18.09	b	20.42	b	53.24	a	46.12	a
Decanoic acid, ethyl ester		92.02	b	119.93	a	112.38	ab	107.01	ab
Diethyl succinate		1605.02	b	1811.84	b	4062.96	a	4285.68	a
1,3-Propanediol diacetate		59.09	b	91.04	a	31.72	c	42.48	c
ethyl 4-hydroxybutanoate		435.89	b	670.47	a	220.22	c	236.90	c
β-phenethyl acetate		213.62	a	252.99	a	134.24	b	144.65	b
Diethyl malate		4956.34	b	7077.68	a	6688.51	ab	7377.40	a
Diethyle 2-hydroxypentanedioate		400.52	c	488.92	bc	1173.09	ab	1231.63	a
Ethyl 5-oxotetrahydro-2-furancarboxylate		732.09	b	1000.19	ab	1144.74	a	1189.90	a
ethyl 2-hydroxy-3-phenylpropanoate		66.00	b	177.16	a	169.67	a	176.19	a

Compound		Sample							
(+)-Diethyl L-tartrate		n.d.		n.d.		1404.67	a	1084.19	a
Ethyl hydrogen succinate		11694.75	b	14260.33	ab	18489.94	ab	22892.76	a
Octadecanoic acid methyl ester		n.d.		n.d.		72.31	b	108.67	a
Pentanedioic acid diethyl ester		108.66	a	114.50	a	157.35	a	144.38	a
Isobutyl phenylacetate		33.05	b	51.02	a	n.d.		n.d.	
	sum	35264.85	c	40332.86	b	50249.97	a	57151.80	a
Others									
Acetoin		264.29	b	314.05	ab	357.89	a	361.96	a
2-Hydroxy-3-pentanone		32.80	b	134.37	a	28.01	b	36.52	b
2-Furaldehyde		n.d.		n.d.		154.89	a	151.58	a
cis-5-hydroxy-2-methyl-1,3-dioxane		25.15	c	33.48	c	80.68	b	109.65	a
2-Methyl-3-thiolannone		n.d.		24.30	a	23.34	a	21.10	a
trans-4-hydroxymethyl-2-methyl-1,3-dioxolane		16.34	c	26.69	b	46.60	a	76.07	a
cis-5-hydroxy-2-methyl-1,3-dioxane		20.62	b	28.44	b	64.53	a	86.50	a
Benzothiazole		19.67	b	29.91	ab	48.01	a	32.39	ab
2,3-dihydroxypyrazine		177.50	c	264.65	bc	403.56	ab	463.75	a
Ethyl 5-oxotetrahydro-2-furancarboxylate		732.08	b	1000.19	ab	1144.73	a	1189.90	a
HMF		n.d.		n.d.		50.98	a	65.46	a
Acetovanillone		33.83	d	49.94	c	189.45	b	234.78	a
	sum	1322.28	c	1939.50	b	2592.67	a	2829.65	a
